# Node-Identification-Based Secure Time Synchronization in Industrial Wireless Sensor Networks

**DOI:** 10.3390/s18082718

**Published:** 2018-08-18

**Authors:** Zhaowei Wang, Peng Zeng, Linghe Kong, Dong Li, Xi Jin

**Affiliations:** 1 Key Laboratory of Networked Control System, Shenyang Institute of Automation, Chinese Academy of Sciences, Shenyang 110016, China; wangzhaowei@sia.cn (Z.W.); lidong@sia.cn (D.L.); jinxi@sia.cn (X.J.); 2 University of Chinese Academy of Sciences, Beijing 100049, China; 3 Department of Computer Science and Engineering, Shanghai Jiao Tong University, Shanghai 200240, China; linghe.kong@sjtu.edu.cn

**Keywords:** time synchronization, industrial wireless sensor networks, security, Sybil attacks, message manipulation attacks, clock skew

## Abstract

Time synchronization is critical for wireless sensors networks in industrial automation, e.g., event detection and process control of industrial plants and equipment need a common time reference. However, cyber-physical attacks are enormous threats causing synchronization protocols to fail. This paper studies the algorithm design and analysis in secure time synchronization for resource-constrained industrial wireless sensor networks under Sybil attacks, which cannot be well addressed by existing methods. A node-identification-based secure time synchronization (NiSTS) protocol is proposed. The main idea of this protocol is to utilize the timestamp correlation among different nodes and the uniqueness of a node’s clock skew to detect invalid information rather than isolating suspicious nodes. In the detection process, each node takes the relative skew with respect to its public neighbor as the basis to determine whether the information is reliable and to filter invalid information. The information filtering mechanism renders NiSTS resistant to Sybil attacks and message manipulation attacks. As a completely distributed protocol, NiSTS is not sensitive to the number of Sybil attackers. Extensive simulations were conducted to demonstrate the efficiency of NiSTS and compare it with existing protocols.

## 1. Introduction

Wireless sensor networks (WSNs) [1] have been extensively employed in cyber physical systems, Internet of Things, and industrial control networks [2,3]. Many applications, including location, data fusion, sleep scheduling, and collaborative operation, require that sensor nodes should have a common notion of time in industrial WSNs (IWSNs) [4]. Each node typically obtains its time by counting the output pulse of the built-in crystal oscillator. However, the dynamic network environment and the heterogeneity of crystal oscillators cause crystal frequency deviations, which renders time asynchronous [5]. The synchronization error is 1.2 ms per minute for a crystal frequency deviation with 20 ppm [6], and cannot satisfy the timing requirement of closed loop control systems. Thus, numerous packet-exchange-based time synchronization protocols and algorithms have been proposed for different application scenarios [6,7,8,9,10,11,12,13,14].

The majority of existing synchronization protocols mainly aim to satisfy common requirements, including high-precision, low-energy consumption, fast synchronization, and robustness for a dynamic network structure. However, security is also an important problem for synchronization protocols [15]. Due to working in unattended environments and the broadcast nature of wireless communication, IWSNs are vulnerable to attacks against physical nodes and communication links [16,17]. These attacks may cause the network to incur invalid time information. Network time synchronization will fail with invalid time information of attackers. Especially for IWSNs in automobile production line, multi-robot cooperation requires high-precision time synchronization. Attacks that break this synchronization may decrease the production efficiency and cause collisions among industrial robots. Thus, traditional protocols cannot be directly applied to hostile environments without any defensive measures.

By investigating current studies [18,19,20,21,22], we learn that many problems remain in designing secure time synchronization mechanisms:

- Centralized time synchronization protocols depend on specific source node and layered topology with poor scalability and robustness. The leaf nodes take their parent nodes as their time sources, and then the branch of the parent nodes will be disabled when the parent nodes are attacked.
- Distributed time synchronization protocols rely on additional clock information to update their clock parameters, e.g., hardware time, logical clock skew, and logical clock offset. Thus, information diversity has created more problems to design secure decentralized time synchronization mechanisms.
- Multiple attack types:. Message manipulation attacks (e.g., replay attack, delay attack, and wormhole attack) and Sybil attacks (e.g., masquerade attack) are two common attack types for sensor networks [18,19,20]. In message manipulation attacks, attackers can modify a time message to interrupt the synchronization process. For Sybil attackers, they pretend to be other nodes and send invalid messages.

The first problem can be solved by adopting a random time source mechanism [23]: a node maintains a time source list and randomly selects its time source based on the quality of the potential time sources. The mechanism enables the node to maintain synchronization with the failure of a fixed time source. To address the second problem, two safeguard mechanisms can be designed for the hardware clock and the logical clock, respectively. Message manipulation attacks in the third problem have been well addressed by current technologies. However, existing secure time synchronization protocols seldom address the Sybil attacks.

Under Sybil attacks, the Sybil attacker in peer to peer networks can forge multiple identities including existing identities and virtual identities, which misleads the valid nodes into establishing a wrong neighbor list [24,25,26]. In combination with sending invalid messages, Sybil attacks can easily attack many protocols in IWSNs, e.g., distributed storage, routing, data aggregation, fair resource allocation, voting, and so on [24]. Therefore, Sybil attacks are one of the most harmful attack types in IWSNs [24,25,26]. Some countermeasures against Sybil attacks have been proposed based on key management [18,27] and neighboring time information [22]. However, they consume more computation, communication, storage, and hardware resources, which are not desirable for resource-constrained IWSNs. For time synchronization under Sybil attacks, if a Sybil attacker sends invalid time messages with different identities, the network synchronization can be disrupted easily. In existing time synchronization protocols against message manipulation attacks [20,21], a safe node disguised by the Sybil attackers would be regarded as a malicious node, and lose synchronization with other nodes. Moreover, due to the scalability and resilience to the node failure of physical attack and Denial of Service attack, decentralized time synchronization protocols are superior to centralized time synchronization protocols. Therefore, we focus on defending the distributed time synchronization protocol against Sybil attacks in this paper.

As indicated in [28], each node has a unique clock skew value that differs from other nodes. Although the authors described the application of clock skew based node identification to detect Sybil attacks, the Sybil attacker may alter its own clock skew by faking timestamps and generating another identity, and then disables the detection. Moreover, it does not focus on time synchronization. Based on our observation, the relative clock skew of two nodes can be calculated by their common neighbor. Hence, we present a novel node-identification-based secure time synchronization (NiSTS) protocol against Sybil attacks. First, each node takes the relative skew with respect to the public neighbor as the judge to filter malicious information rather than shielding the suspicious nodes and then updates its clock with valid messages. The main contributions of this paper are summarized as follows:

(1) Based on the theoretical and simulation analysis, we prove that the logical clock error of the secured maximum consensus based time synchronization (SMTS) [20] protocol under Sybil attacks linearly increases eventually with probability.
(2) Based on the timestamp correlation among different nodes and the uniqueness of a node’s clock skew, we utilize a relative skew checking mechanism to filter the fallacious messages in the design of NiSTS.
(3) We provide the effectiveness analysis of NiSTS, and the performance comparison in terms of energy consumption and security. Simulation results are conducted to prove the effectiveness of NiSTS.

The remainder of the paper is organized as follows: Section 2 presents related work. In Section 3, we briefly describe the system model and problem formulation. Section 4 details the NiSTS protocol. Simulation is presented to validate the efficiency of the proposed protocol in Section 5. Section 6 concludes the paper.

## 2. Related Work

There have been numerous studies for secure time synchronization in WSNs. We divide the existing literature into two categories based on the network structure: countermeasures for attacks in centralized synchronization and distributed synchronization.

### 2.1. Countermeasures for Attacks in Centralized Synchronization

All sensor nodes synchronize their clock to a reference source in centralized synchronization [7,8,9,10]. Many studies have been performed to provide countermeasures for centralized synchronization in hostile environment [18,27,29,30,31,32,33].

Sun et al. [29] proposed two resilient clock synchronization schemes based on level and diffusion, respectively. The schemes adopt the median of multiple source clock differences as the source clock difference and can handle various attacks. However, the proposed schemes cannot defend against attacks on the timeliness of synchronization messages, e.g., delay and wormhole attacks. Using the hardware-assisted, authenticated media access control (MAC) layer timestamping and the *μ*TESLA broadcast authentication protocol, the authors in [30] developed the TinySeRsync for WSNs that run TinyOS in a subsequent study. Du et al. [18] presented secure and efficient time synchronization for heterogeneous sensor networks. Public key cryptography (PKC) is utilized to prevent various attacks. Because all nodes need to store the keys of other nodes, the technique requires additional communication and storage overhead. To reduce the overhead of each node, Rahman et al. [31] employed pairing-based cryptography to secure the time synchronization protocol for both homogeneous WSNs and heterogeneous WSNs. By checking whether the end-to-end delay exceeds the preset maximal expected message delay, Ganeriwal et al. [32] proposed a suite of protocols for secure pairwise and group synchronization to defend against the delay attack. Using a PKC-based authentication scheme, random nonce, and threshold-based delay attack detection, the secure pairwise broadcast synchronization protocol of Benzaid et al. [27] is resilient to almost all attacks. As opposed to cryptographic countermeasures, Roosta et al. [33] proposed robust regression-method-based statistical countermeasures to design a secure flooding time synchronization protocol.

### 2.2. Countermeasures for Attacks in Distributed Synchronization

In distributed synchronization, without a special topology, each node uses their neighbor’s clock information to realize synchronization with each other [6,11,12,13]. Some secure distributed synchronization protocols have also been proposed [20,21,22,34,35].

Sun et al. [34] proposed a fault-tolerant time synchronization protocol for cluster-wise clock synchronization in WSNs. The protocol exploits a symmetric cryptography-based local broadcast authentication technique. However, the protocol requires that the nodes in a cluster should maintain initial synchronization. Moreover, the cluster consists of fully connected nodes. Unlike cryptographic countermeasures, timestamp correlation among different nodes is employed to check whether a neighbor’s clock information is reliable. This technology frequently appears in the design of secure distributed synchronization [20,21,22,35]. Hu et al. [35] exploited the high temporal correlation among adjacent nodes, i.e., the linearity of the hardware clock, to design attack tolerant time synchronization. However, the clock skew is not compensated. To compensate both clock offset and skew, He et al. [21] proposed secure average-consensus-based time synchronization against message manipulation attacks. The checking mechanism in [21] includes two parts: the hardware clock checking process and the logical clock checking process. Similar to the study in [21], a novel secured maximum-consensus-based time synchronization (SMTS) protocol is presented by He et al. [20]. Recently, Dong et al. [22] developed a robust and secure time synchronization protocol to defend against Sybil attacks. The core idea is based on a graph theoretical approach to perform anomaly detection at the message level. However, additional storage overhead with $O\left(M^{2}\right)$ time complexity is required, where *M* is the number of the most recent time synchronization messages for each neighbor.

These countermeasures for various attacks in centralized and distributed synchronization rely on traditional cryptography, statistics, or a threshold-based technique. However, they seldom consider Sybil attacks. Under the Sybil attacks, a malicious device can illegitimately take on multiple identities [24]. Time synchronization methods will be disabled because some safe nodes are recognized as the attackers under Sybil attacks. Although Dong et al. [22] proposed a resilient synchronization method against Sybil attacks, additional communication, storage, and computation resources are required for resource-constrained IWSNs. In addition, because centralized synchronization methods depend on fixed source nodes and a hierarchical structure, they are more vulnerable to the single point of failure and Sybil attacks. Due to the low overhead and expandability of IWSNs, NiSTS builds on top of distributed synchronization against Sybil attacks.

## 3. System Model and Problem Formulation

First, we define the network model, clock model and attack model that are involved in the problem before introducing the proposed method. Second, we formulate the problem.

### 3.1. Network Model

Consider an IWSN without a time source but with *n* safe nodes and *m* attackers in the network, where n>m. To accelerate the synchronization process, each node maintains a sufficient number of neighbors using two approaches. For a target area, we can increase the transmission power of each node and the number of nodes. Because all nodes are equal communication entities, a bidirectional neighbor relationship exists. Let an undirected graph G=(V,E) denote an IWSN, where V={1,2,⋯,n+m} is the set of nodes, and (i,j)∈E indicates that node *i* and node *j* can communicate with each other. $V_{s}=\left\{1,2,\cdots ,n\right\}$ is the set of safe nodes. $N_{i}=\left\{j|j\in V,e_{ij}\in E\right\}$ denotes the neighbors of Node *i*. Each node has more than one neighbor. At least a common neighbor exists between any node *i* and node *j*, i.e., $N_{i}\cap N_{j}\neq \emptyset$, $j\in N_{i}$. Some important notation definitions are listed in Table 1.

### 3.2. Clock Model

Each sensor node in IWSNs is equipped with an internal crystal oscillator, which periodically outputs the pulse based on the crystal frequency. A counter register is employed to store the number of the output pulse. Thus, a node’s time can be obtained from its counter register. A first-order linear clock model is extensively applied to describe the timing process [12,13], namely, the hardware clock $\tau_{i}\left(t\right)$ of node *i* is

(1) $$\tau_{i}\left(t\right)=\alpha_{i}t+\beta_{i},$$

where $\tau_{i}\left(t\right)$ is an increasing function of absolute time *t*, and $\alpha_{i}$ and $\beta_{i}$ are the hardware clock skew and offset, which determines the timing rate and the initial synchronization error, respectively. The hardware clock skew $\alpha_{i}$ depends on the hardware manufacturing process and the environment interference, which causes the clock skew to dynamically deviate from 1, i.e., $1-\rho \leq \alpha_{i}\leq 1+\rho$, where ρ is typically on the order of 5–20 ppm [6]. However, the skew changes very slowly over a short period; thus, it is static during one sync interval. In general, the initial time is the start-up time of each sensor node. We define the initial time as $t_{0}$, namely, $t_{0}=0$. Thus, the hardware clock offset $\beta_{i}$ is the offset of node *i* at time $t_{0}$, namely, $\beta_{i}=\tau_{i}\left(0\right)$. Since the absolute time is not available, we cannot directly modify the clock skew and offset to compensate the hardware clock. To modify the time, we establish the logical clock ${\hat{\tau }}_{i}\left(t\right)$ of node *i* [12,13], which is given by

(2) $${\hat{\tau }}_{i}\left(t\right)={\hat{\alpha }}_{i}\left(t\right)\tau_{i}\left(t\right)+{\hat{\beta }}_{i}\left(t\right)={\hat{\alpha }}_{i}\left(t\right)\alpha_{i}t+\left({\hat{\alpha }}_{i}\left(t\right)\beta_{i}+{\hat{\beta }}_{i}\left(t\right)\right),$$

where ${\hat{\alpha }}_{i}\left(t\right)$ and ${\hat{\beta }}_{i}\left(t\right)$ are the logical clock skew and offset compensation parameters at time *t*, respectively, which can be updated via time synchronization algorithms.

### 3.3. Attack Model

Time synchronization protocols in IWSNs are vulnerable to different attacks, since IWSNs are usually deployed in an untended environment. In this paper, we primarily focus on the defense of distributed time synchronization protocol against Sybil attacks. In [24], the authors considered Sybil attacks as a malicious device that illegitimately takes on multiple identities. Here, we assume that a Sybil attacker can only disguise itself as its neighbor. At the same time, invalid messages cannot be relayed in a multi-hop manner.

At the initialization phase, each sensor node exchanges identity information with adjacent nodes to construct a fully distributed IWSN. Sybil attackers in an IWSN can take on multiple identities, which are obtained in two ways: neighbor identities and self-created new identities. The random self-created identity can easily be distinguished by a threshold as each node sends its time messages in a predefined and limited period. This situation has been extensively studied and is not repeated in our work. Moreover, when there are two or more consecutive messages with same self-created identity, this attack type can be considered as the message manipulation attack. Considering the resource constraint in IWSNs, in distributed synchronization, each node uses the messages sent by direct neighbors to calibrate the clock, and the received messages would not be further forwarded to other nodes. Thus, the above assumption is reasonable.

**Figure 1:**
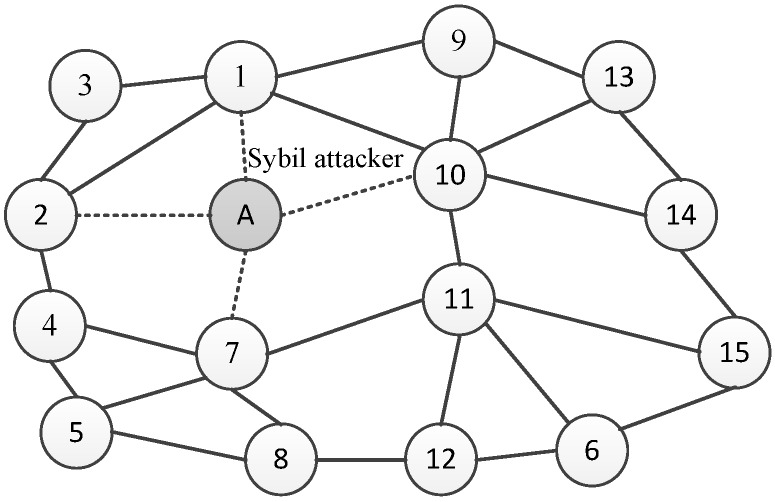
IWSNs with a Sybil attacker.

For example, in Figure 1, node *A* is the Sybil attacker. Node *A* has four neighbor nodes, i.e., Nodes 1, 2, 7, and 10. In each synchronization cycle, attacker *A* broadcasts fake messages with an identity that is randomly selected from Nodes 1, 2, 7, and 10. The fake messages can only be heard by the neighbor of attacker *A*, and would not be spread to further nodes.

**Definition** **1.** *When a safe node is disguised by the Sybil attacker, it is called suspicious node.*

### 3.4. Problem Formulation

In a typical distributed time synchronization protocol, e.g., the Average TimeSync (ATS) protocol [12] and the Maximum Time Synchronization (MTS) protocol [13], each node updates the logical clock compensation parameters ${\hat{\alpha }}_{i}$ and ${\hat{\beta }}_{i}$ based on the received synchronization messages $<\tau_{i}\left(t\right),{\hat{\alpha }}_{i}\left(t\right),{\hat{\beta }}_{i}\left(t\right)>$ from its neighbor. The objectives of ATS and MTS are to make the network’s clock equal to the average value and the maximum value of all nodes’ logical clocks, respectively. However, synchronization cannot be realized with a wrong synchronization message and an invalid neighbor.

Therefore, the purpose of this paper is to design a secure clock synchronization protocol to obtain valid messages among messages with same identity, such that

(3) $$\lim_{t\to \infty }{\hat{\tau }}_{i}\left(t\right)=\lim_{t\to \infty }{\hat{\tau }}_{j}\left(t\right),\forall i,j\in V_{s}.$$

## 4. NiSTS Protocol

Since all possible attacks cannot be prevented for all types of protocols, we would like to make the distributed synchronization protocol resilient to Sybil attacks. The proposed NiSTS consists of two parts: the detection process to filter malicious messages and the clock update process to realize time synchronization. Next, we detail the detection process, the design of NiSTS with the update rules, and the performance analysis.

### 4.1. Detection Process

Huang et al. [28] pointed out that the clock skew error between each node and a fixed node is different and stable. In [28], normal nodes synchronize their time with the fixed node, i.e., the root node *r*. The clock skew error $\alpha_{i}-\alpha_{r}$ is determined to be unique based on the observation in physical experiment. Obviously, the clock skew error $\alpha_{i}-1$ between the node *i* and the absolute time *t* is also unique. Thus, the stability and the uniqueness of the clock skew is valid in our study, and the relative clock skew of two nodes is constant.

Based on this observation, we utilize the uniqueness of relative clock skew between two nodes to detect the malicious messages. The relative clock skew is defined as

(4) $$\alpha_{ij}\left(k\right)=\frac{\tau_{j}\left(t_{k}\right)-\tau_{j}\left(t_{k-1}\right)}{\tau_{i}\left(t_{k}\right)-\tau_{i}\left(t_{k-1}\right)},$$

where *k* denotes the *k*th iteration. The message with identity *j* is valid if and only if $\alpha_{ij}\left(k\right)=\alpha_{ij}\left(k-1\right)$. Under Sybil attacks, node *i* cannot confirm the real $\alpha_{ij}$ with mixed messages, which include valid messages and invalid messages. However, node *i* can obtain the real $\alpha_{jc}$ from node *c*, who is a credible common neighbor of node *i* and node *j*. Therefore, the real $\alpha_{ij}$ should satisfy

(5) $$\alpha_{ij}\left(k\right)\cdot \alpha_{jc}\left(k\right)=\frac{\tau_{j}\left(t_{k}\right)-\tau_{j}\left(t_{k-1}\right)}{\tau_{i}\left(t_{k}\right)-\tau_{i}\left(t_{k-1}\right)}\cdot \frac{\tau_{c}\left(t_{k}\right)-\tau_{c}\left(t_{k-1}\right)}{\tau_{j}\left(t_{k}\right)-\tau_{j}\left(t_{k-1}\right)}=\alpha_{ic}\left(k\right).$$

Now, node *i* can utilize the detection process to filter the invalid messages.

**Figure 2:**
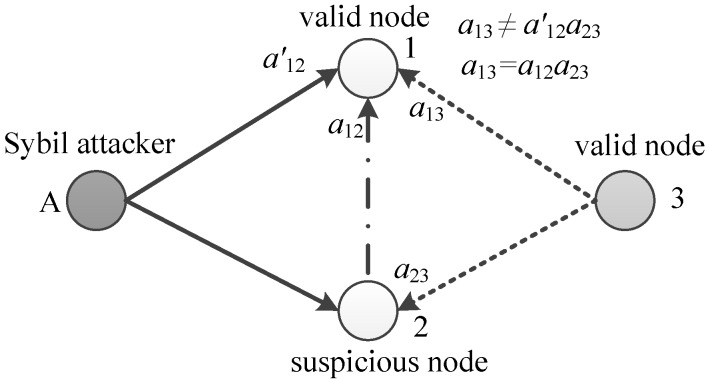
Illustration of the detection process. Attacker *A* pretends to be Node 2.

**Figure 3:**
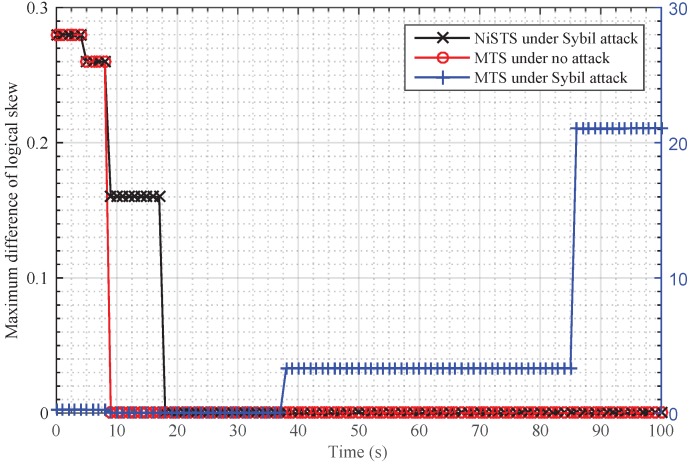
Performance of the detection process under the Sybil attack.

We use an example in Figure 2 to illustrate the detection process. Consider a simple network with four nodes, where node *A* is the Sybil attacker. Node *A* can disguise itself as Nodes 1 and 2 to broadcast invalid messages, and Node 3 is the common neighbor of the two nodes. In Figure 2, we assume that the Sybil attacker pretends to be Node 2. Node 1 can receive valid messages from Nodes 2, 3 and invalid messages from node *A*, but cannot distinguish whether the message with identity 2 is valid. In our detection process, Node 1 and Node 2 calculate the relative skew $\alpha_{13}$ and $\alpha_{23}$ with real messages. Then, Node 1 evaluates whether the condition $\alpha_{13}=\alpha_{12}\cdot \alpha_{23}$ is established. The condition is used to detect the invalid message from the Sybil attacker since it does not satisfy the equation, namely, the uniqueness of relative clock skew. Figure 3 shows the effectiveness of our detection process. The blue line corresponds to the right ordinate. Our protocol NiSTS can achieve synchronization of the logical clock skew ${\hat{\alpha }}_{i}\alpha_{i}$ under Sybil attacks.

In [20,21], Node 2 is directly regarded as a malicious node when Node 1 receives suspicious messages from Sybil attacker *A* with identity 2, which is unfair for Node 2. Under the relative skew estimation (RSE) algorithm in SMTS [20], the Sybil attacks would disable the communication between Node 1 and Node 2. However, it should be noted that the synchronization in [20] can also be achieved here as the synchronous path along Nodes 1, 3, and 2 is not influenced. When Node 3 faces a disguised threat, the communication link along Nodes 1, 3, and 2 would lose efficacy, namely, SMTS is useless under Sybil attacks. More details on why SMTS fails under Sybil attacks are given in Section 4.3.2. Figure 2 is a simple example to illustrate our detection process. Unlike SMTS, our detection process continues to process the following messages of suspicious nodes instead of breaking up with the suspicious nodes, which increases the synchronization probability of suspicious nodes. The effectiveness of the detection process is detailed in Section 4.3.1.

### 4.2. NiSTS Protocol Design

The maximum value of the clock parameters can easily be acquired via one comparison but the acquisition of the average value requires multiple iterations. Thus, we develop the secure time synchronization protocol based on the MTS protocol. The pseudo code of NiSTS protocol is described in Algorithm 1.

**Algorithm 1** NiSTS protocol.**Input:**G=(V,E), ${\hat{\alpha }}_{i}=1$, ${\hat{\beta }}_{i}=0$, *T*, $\alpha_{ij}=1$, ∀i,j∈V, $e_{ij}\in E$, $j\in N_{i}$.**Output:**${\hat{\alpha }}_{i}$, ${\hat{\beta }}_{i}$, ∀i∈V.1. ∀i∈V, if $\tau_{i}\left(t\right)=kT,k\in N^{+}$, node *i* broadcasts $\langle \tau_{i}\left(t\right),{\hat{\alpha }}_{i}\left(t\right),{\hat{\beta }}_{i}\left(t\right)\rangle$ and $\alpha_{ij},j\in N_{i}$.2. Upon receiving time information from node *j*, node *i* records $\langle \tau_{i}\left(t_{k}\right),{\hat{\alpha }}_{i}\left(t_{k}\right),{\hat{\beta }}_{i}\left(t_{k}\right)\rangle$.3. Node *i* estimates $\alpha_{ij}$ with (4) when it has a historical record $\langle \tau_{j}\left(t_{k-1}\right),{\hat{\alpha }}_{j}\left(t_{k-1}\right),{\hat{\beta }}_{j}\left(t_{k-1}\right)\rangle$.4. If $\alpha_{jc}=1$ or $\alpha_{ic}=1$, $c\in N_{i}\cap N_{j}$:      node *i* stores $\alpha_{ij}$ without any updating.   else if $\alpha_{ij}\cdot \alpha_{jc}=\alpha_{ic}$:      node *i* stores $\left(\tau_{i}\left(t_{k}\right),\tau_{j}\left(t_{k}\right)\right)$, $\alpha_{ij}$ and updates $\langle {\hat{\alpha }}_{i},{\hat{\beta }}_{i}\rangle$.

In our protocol, each node maintains a neighbor repository to store the latest time information about its neighbor, including the logical clock skew and offset compensation parameters, hardware time, and the relative clock skew with respect to its neighbor. When each node is powered on, the time information of each node must be initialized, namely, set ${\hat{\alpha }}_{i}=1$, ${\hat{\beta }}_{i}=0$, and $\alpha_{ij}=1$. If the current hardware time of node *i* satisfies the condition $\tau_{i}\left(t\right)=kT,k\in N^{+}$, where *T* is the predefined broadcast period, *i* broadcasts its current time information $\langle \tau_{i}\left(t\right),{\hat{\alpha }}_{i}\left(t\right),{\hat{\beta }}_{i}\left(t\right)\rangle$ and $\alpha_{ij},j\in N_{i}$. At each iteration, when node *i* receives a time message from its neighbor *j*, it records its logical clock skew and offset compensation parameters and the hardware clock reading. If it has a historical record of node *j*, then it computes the relative skew $\alpha_{ij}$.

In the detection process, node *i* checks whether $\alpha_{ij}\cdot \alpha_{jc}$ is equal to $\alpha_{ic}$, where *c* is the common valid neighbor of node *i* and node *j*. If $\alpha_{ij}\cdot \alpha_{jc}=\alpha_{ic}$, the time message with identity *j* is valid, and node *i* will update their logical clock parameters with the valid message. The update rules are described as follows:

If ${\hat{\alpha }}_{i}\left(t_{k}\right)<\alpha_{ij}\left(k\right){\hat{\alpha }}_{j}\left(t_{k}\right)$:

(6) $${\hat{\alpha }}_{i}\left(t_{k}^{+}\right)\leftarrow \alpha_{ij}\left(k\right){\hat{\alpha }}_{j}\left(t_{k}\right),$$

(7) $${\hat{\beta }}_{i}\left(t_{k}^{+}\right)\leftarrow {\hat{\alpha }}_{j}\left(t_{k}\right)\tau_{j}\left(t_{k}\right)+{\hat{\beta }}_{j}\left(t_{k}\right)-{\hat{\alpha }}_{i}\left(t_{k}\right)\tau_{i}\left(t_{k}\right).$$

else if ${\hat{\alpha }}_{i}\left(t_{k}\right)=\alpha_{ij}\left(k\right){\hat{\alpha }}_{j}\left(t_{k}\right)$:

(8) $${\hat{\beta }}_{i}\left(t_{k}^{+}\right)\leftarrow \max_{l=i,j}\left({\hat{\alpha }}_{l}\left(t_{k}\right)\tau_{l}\left(t_{k}\right)+{\hat{\beta }}_{l}\left(t_{k}\right)\right)-{\hat{\alpha }}_{i}\left(t_{k}\right)\tau_{i}\left(t_{k}\right).$$

Since each node periodically transmits the time information at a predefined interval *T* based on its hardware clock, the absolute transmission time of node *i* is tki=kT−βikT−βiαiαi,k∈N+. Each hardware clock skew $\alpha_{i}$ is different [6], and the value of βiβiαiαi is constant. Thus, each $t_{k}^{i}$ is different, i.e., the transmission mechanism of time information is asynchronous transmission. Asynchronous transmission avoids the channel collision and reduces the communication overhead. At each iteration, each node synchronizes its logical clock to the faster clock of its neighbor clocks based on the update rules. The global time synchronization is achieved with Equation (3).

Here, MAC layer time stamping is used to eliminate the uncertain delay in the sensor nodes [12,14], and the distance between two neighbor nodes is limited. Thus, we assume that the communication delays are constant.

### 4.3. Performance Analysis

In this subsection, we analyze the effectiveness of the detection process, and detail the performance comparison.

#### 4.3.1. Analysis of the Detection Process

In the detection process, when two nodes confirm the validity of the messages from each other, they need to rely on a valid common neighbor. Thus, the number of common neighbors influences the performance of the NiSTS protocol. We employ a theoretical analysis to illustrate the effectiveness of the NiSTS protocol.

**Figure 4:**
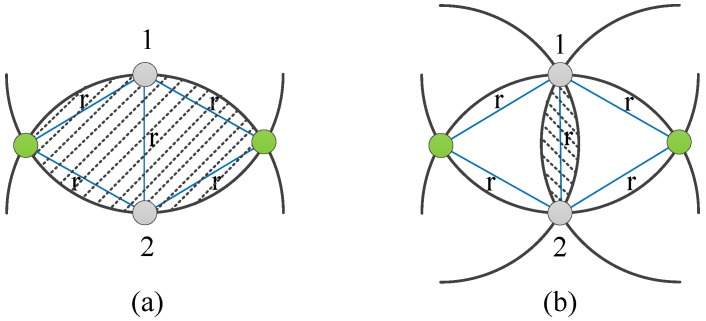
Illustration of the effectiveness of the detection process: (**a**) area with common neighbors; and (**b**) area with Sybil attackers that may disable the detection process.

Consider an IWSN with *n* safe nodes and *m* Sybil attackers. All nodes are randomly deployed over an l×l square area. The communication range of each node is set to *r*. As shown in Figure 4a, the nodes in the shadow area can hear the messages from the two adjacent Nodes 1 and 2 with the farthest distance *r*. The green nodes are the two farthest common neighbors of Nodes 1 and 2. Thus, the minimum number of common neighbors of any two adjacent nodes is $\frac{nr^{2}}{l^{2}}\left(\frac{2\pi }{3}-\frac{\sqrt{3}}{2}\right)\approx 1.23\frac{nr^{2}}{l^{2}}$. In the worst-case scenario, all common neighbors are suspicious nodes, and the detection process in NiSTS risks failure. As shown in Figure 4b, Sybil attackers in the shadow area can disguise themselves as all common neighbors of Nodes 1 and 2. The number of Sybil attackers in the shadow area is $\frac{mr^{2}}{l^{2}}\left(\frac{\pi }{3}-\frac{\sqrt{3}}{2}\right)\approx 0.18\frac{mr^{2}}{l^{2}}$.

We develop the following equation:

(9) $$0.18\frac{mr^{2}}{l^{2}}\leq \varepsilon ,$$

where ε is a probability factor that indicates the effectiveness of NiSTS. When ε≤0.1, the reliability of NiSTS can exceed 90% probability. At this time, the number of Sybil attackers may be limited to $0.56\frac{l^{2}}{r^{2}}$. Thus, the NiSTS protocol can defend against more Sybil attackers with a larger network area and a less communication power.

Next, we detail the probability analysis of the proposed NiSTS protocol risking failure in the worst-case scenario. The detection process is effective under the condition that at least a credible common neighbor exists between Node 1 and Node 2, as shown in Figure 4. During the current detection process $\alpha_{12}\cdot \alpha_{2c}=\alpha_{1c}$, $c\in N_{1}\cap N_{2}$, the common node *c* is credible means that the relative clock skews $\alpha_{1c}$ and $\alpha_{2c}$ are both valid. Meanwhile, the faked identity of a Sybil attacker is randomly selected from the attacker’s neighbor, namely, the probability that node *c* is disguised during one period is $\frac{l^{2}}{1.23nr^{2}}$ by an attacker. As the relative clock skew is calculated by using two consecutive time messages and the number of Sybil attackers in the worst-case scenario is $0.18\frac{mr^{2}}{l^{2}}$, the probability that the common node *l* is credible during two consecutive periods is $\sigma ={\left(1-\frac{l^{2}}{1.23nr^{2}}\right)}^{2\cdot \frac{0.18mr^{2}}{l^{2}}}$. When all the common nodes are disguised during these two consecutive periods, the detection process is out of work. Thus, the probability is ${\left(1-\sigma \right)}^{\frac{1.23nr^{2}}{l^{2}}}$ for $\frac{1.23nr^{2}}{l^{2}}$ common neighbors.

As presented in the previous section, $0.18\frac{mr^{2}}{l^{2}}\leq \varepsilon \leq 0.1$, it is obvious that σ≅1 and 1−σ≪1. Hence, the probability is ${\left(1-\sigma \right)}^{\frac{1.23nr^{2}}{l^{2}}}\ll 1$ that the current detection process fails in a dense network. Because NiSTS algorithm does not isolate the suspicious nodes, the detection process will not be interrupted. Therefore, with the increasing of the iterations *N*, the failure probability of the algorithm approaches 0, i.e., $\lim_{N\to +\infty }{\left(1-\sigma \right)}^{\frac{1.23nr^{2}}{l^{2}}\cdot N}=0$. The validity of the algorithm is proven indirectly. Hence, the NiSTS protocol will be more effective in a dense network with more common neighbors.

#### 4.3.2. Performance Comparison

Low-energy and security are key technologies for IWSNs that are usually working in an unattended environment. It is well known that the computational energy cost of MTS based updating algorithms is comparably small [13], and the communication energy consumption occupies main energy cost in sensor nodes [4]. Moreover, the nodes exchange messages frequently in distributed timesync protocols. Hence, the communication energy cost, which can be analyzed by the broadcasting times [4,13,20], is an intuitive illustration of the protocol energy consumption. As the nodes broadcast local clock information periodically at a given time interval, the broadcasting times is proportional to the convergence time of the timesync protocol, and then a shorter convergence time reveals a lower energy consumption. The energy consumption can be further analyzed by the convergence time of protocols. Therefore, in this subsection, we focus on the performance comparison of the convergence time and the security of NiSTS with the performance of the convergence time and the security of MTS and SMTS in three cases: no attack, message manipulation attacks and Sybil attacks. Here, time synchronization refers to the synchronization of safe nodes.

*Case 1*: **Under no attack**. Based on the previous analysis, MTS is effective in a benign environment, with the convergence time $T_{con}^{MTS}\leq B\left(n-1\right)$ [13], where *B* denotes that the message broadcast occurs twice during the period, i.e., MTS updates its clock parameters with each $\alpha_{ij}\left(k\right)$, k≥1. SMTS achieves time synchronization with the verification mechanism $\alpha_{ij}\left(k\right)=\alpha_{ij}\left(1\right)$, k≥2. NiSTS realizes synchronization with the message filtering process $\alpha_{ij}\left(k\right)=\alpha_{ic}\left(k\right)\cdot \alpha_{cj}\left(k\right)$, i.e., NiSTS starts with at least three relative clock skews, where k≥3. NiSTS needs additional time information about the neighbors to start. Thus, the convergence time of MTS is the shortest, and the convergence time of NiSTS is the longest.

*Case 2*: **Under message manipulation attacks**. A message manipulation attacker broadcasts the error hardware clock reading to its neighbors. Thus, the relative clock skews that are computed by the receiving nodes are invalid. In [20], the authors presented a detailed theoretical and simulation analysis to illustrate that MTS would be invalid under message manipulation attacks; thus, we do not repeat it here. In SMTS, the linear clock model is utilized to check the consecutiveness of the neighbor hardware clocks at each iteration. Each node shields the attackers when $\alpha_{ij}\left(k\right)\neq \alpha_{ij}\left(1\right)$, k≥2. Since the detection process of NiSTS is also based on the valid relative clock skew, NiSTS is also valid under manipulation attacks with a longer convergence time.

*Case 3*: **Under Sybil attacks**. A Sybil attacker randomly pretends to be its neighbor to advertise falsified time information. Similarly, MTS is invalid under Sybil attacks. In SMTS, the messages from suspicious node could not pass the verification process $\alpha_{ij}\left(k\right)=\alpha_{ij}\left(1\right)$, k≥2. The safeguard mechanism will deem the node as an attacker, and break up with the node by ignoring all its following messages. In fact, the suspicious node is a safe node that needs synchronization. Hence, under Sybil attack, SMTS is disabled and renders the networks out of synchronization with probability, which is given by the following theorem.

**Theorem** **1.** *The logical clock error of SMTS under Sybil attacks linearly increases at last with probability, i.e.,*

(10) $$\Delta \hat{\tau }\left(t\right)=At+C,t\geq D$$

*where, D is a time constant, and A and C are the maximum differences of the logical clock skew and the logical clock offset of the safe nodes, respectively.*

**Figure 5:**
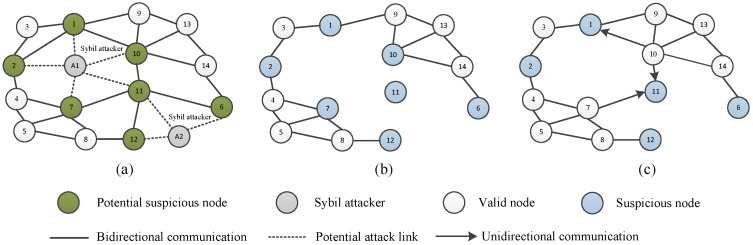
Illustration of the network scenarios for SMTS under Sybil attacks: (**a**) a network with two Sybil attackers; (**b**) at least one isolated node exists; and (**c**) no isolated nodes exist.

**Proof** **of** **Theorem** **1.** From Definition 1 of the suspicious node, we divide the safe nodes of WSNs into valid nodes and suspicious nodes. Let $V_{valid}$, $V_{sus}$, and $V_{iso}$ denote the sets of valid nodes, suspicious nodes, and isolated nodes, respectively. As shown in Figure 5a, the neighbors of the Sybil attackers are called potential suspicious nodes. These potential suspicious nodes become real suspicious nodes when they are disguised. Due to the safeguard mechanism in SMTS, the communication link is interrupted when two nodes are both suspicious nodes. For example, Node 1 and Node 2 cannot communicate with each other, as shown in Figure 5b. For two potential suspicious nodes, their communication becomes a unidirectional communication when only one node is a suspicious node, as shown in Figure 5c. Hence, the communication network of SMTS under Sybil attacks consists of two types. Figure 5b,c shows the pruned networks when suspicious nodes exist. We analyze the synchronization process of SMTS under Sybil attacks in these two scenarios.

(1) At least one isolated node, which cannot exchange messages with other nodes, exists, as shown in Figure 5b. Thus, the clock information about the isolated nodes remains unchanged, and the maximum differences of the logical skew and the logical offset of the safe nodes are $A=\max_{q\in V_{iso},i\in \left\{V_{s}-V_{iso}\right\}}\left\{{\hat{\alpha }}_{q}\left(t\right)\alpha_{q}-{\hat{\alpha }}_{i}\left(t\right)\alpha_{i}\right\}$ and $B=\max_{q\in V_{iso},i\in \left\{V_{s}-V_{iso}\right\}}\left\{{\hat{\alpha }}_{q}\left(t\right)\beta_{q}+{\hat{\beta }}_{q}\left(t\right)-(\left({\hat{\alpha }}_{i}\left(t\right)\beta_{i}+{\hat{\beta }}_{i}\left(t\right)\right)\right\}$, respectively.
(2) No isolated nodes exist. However, since suspicious nodes cannot pass the safeguard process of SMTS, a suspicious node can only receive messages but cannot transmit messages, as shown in Figure 5c. The valid nodes easily achieve synchronization. For suspicious nodes, when $\alpha_{j}=\max_{i\in V_{s}}\left\{\alpha_{i}\right\},j\in V_{valid}$, suspicious nodes can synchronize the valid nodes based on Equations (6)–(8). When $\alpha_{q}=\max_{i\in V_{s}}\left\{\alpha_{i}\right\},q\in V_{sus}$, the clock state of node *q* remains unchanged. Therefore, the maximum differences of the logical skew and offset of the safe nodes are $A=\max_{q\in V_{sus},i\in V_{valid}}\left\{{\hat{\alpha }}_{q}\left(t\right)\alpha_{q}-{\hat{\alpha }}_{i}\left(t\right)\alpha_{i}\right\}$ and $B=\max_{q\in V_{sus},m\in V_{valid}}\left\{{\hat{\alpha }}_{q}\left(t\right)\beta_{q}+{\hat{\beta }}_{q}\left(t\right)-\left({\hat{\alpha }}_{m}\left(t\right)\beta_{m}+{\hat{\beta }}_{m}\left(t\right)\right)\right\}$, respectively.

Here, *D* denotes the synchronization time of the safe nodes. The logical clock error of SMTS increases linearly after the constant time *D*. Therefore, the theorem holds. ☐

Unlike SMTS, our protocol NiSTS filters the invalid messages instead of isolating the suspicious nodes. The above analysis reveals that the synchronization process of NiSTS is the same under no attack, manipulation attacks, and Sybil attacks. Thus, NiSTS is not sensitive to attack types. Nevertheless, NiSTS would increase the communication overhead with longer convergence time.

Compared with [22], NiSTS does not need to allocate additional memory space to store the most recent *M* time messages for each neighbor. In the detection process of NiSTS, it just needs to store the latest time message and assesses whether the received message is valid based on Equation (5). As there are multiple common neighbors between the node *i* and its neighbor *j*, NiSTS can distinguish an invalid message with a maximum of $|N_{i}\cap N_{j}|$ times multiplication. For [22], the time complexity is $O\left(M^{2}\right)$.

## 5. Evaluation

In this section, we present simulations to evaluate our method under different attack types in comparison with MTS [13] and secured maximum-consensus-based time synchronization (SMTS) [20]. The following simulations were conducted in Matlab R2015a.

We considered an IWSN with 30 nodes, which are randomly deployed in a 1×1 area. The parameters of each node were set as follows [13,20]: the communication range is $\sqrt{0.1}$; the logical skew and offset compensation parameters are 1 and 0 s, respectively; the clock skew and offset are randomly selected from 0.8–1.2 and 0–0.4 s, respectively; and the broadcast period *T* is 1 s. Additionally, each node is equipped with a 32.768 kHz crystal oscillator. The probability factor ε is 0.1. Hence, the maximum number of Sybil attackers in this IWSN is 5. We applied the maximum difference of the logical clock skew ${\hat{\alpha }}_{i}\alpha_{i}$ and the maximum difference of the logical clock offset ${\hat{\alpha }}_{i}\beta_{i}+{\hat{\beta }}_{i}$ to represent the synchronization precision. All safe nodes achieve synchronization if and only if $\max_{i,j\in V_{s}}\left\{\left({\hat{\alpha }}_{i}\beta_{i}+{\hat{\beta }}_{i}\right)-\left({\hat{\alpha }}_{j}\beta_{j}+{\hat{\beta }}_{j}\right)\right\}=0$ and $\max_{i,j\in V_{s}}\left\{{\hat{\alpha }}_{i}\alpha_{i}-{\hat{\alpha }}_{j}\alpha_{j}\right\}=0$. Since the logical clock skew and offset simultaneously update, they also converge synchronously.

**Figure 6:**
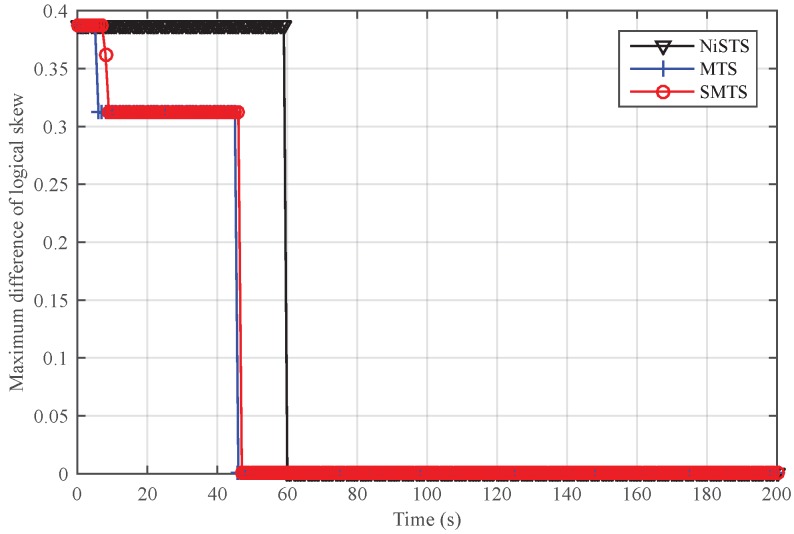
Maximum difference of logical skew in a reliable environment.

**Figure 7:**
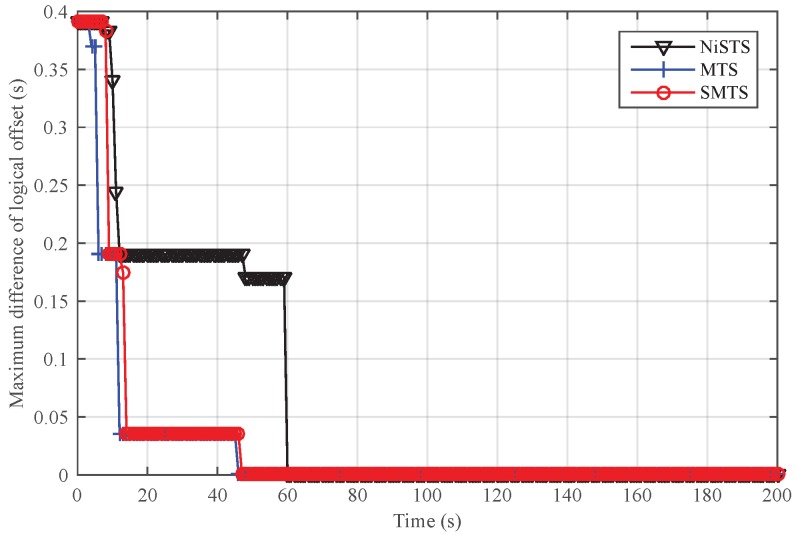
Maximum difference of logical offset in a reliable environment.

First, we applied these protocols in a reliable environment. Figure 6 and Figure 7 demonstrate that all protocols could achieve logical clock skew and offset synchronization simultaneously in a reliable environment because all nodes are valid. As the update rules are same for MTS, SMTS, and NiSTS protocols, the convergence time of each protocol is determined by the updating start time of logical clock parameters. Furthermore, each protocol starts updating based on the computed relative clock skew $\alpha_{ij}$. For MTS, it starts the updating once obtaining one relative clock skew with one neighbor, namely, $\alpha_{ij}\left(k\right)$, k≥1. SMTS adds a detection process to check whether the received messages is valid before updating, namely, $\alpha_{ij}\left(k\right)=\alpha_{ij}\left(1\right)$, k≥2. For NiSTS, it adopts three relative clock skews to detect invalid messages before updating, namely, $\alpha_{ij}\left(k\right)=\alpha_{ic}\left(k\right)\cdot \alpha_{cj}\left(k\right)$, k≥3. Therefore, the convergence rate of MTS is the fastest; conversely, NiSTS’s convergence rate is the slowest. Because the logical clock skew and offset converge simultaneously, we employed the logical clock skew to denote the synchronization performance in part of the following simulation analysis.

**Figure 8:**
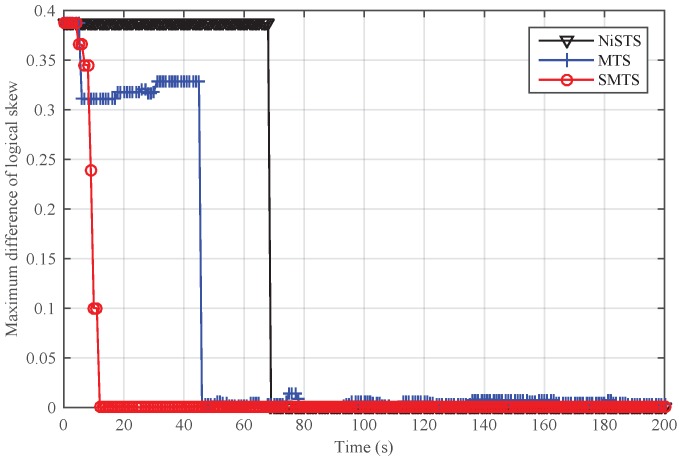
Maximum difference of logical skew under message manipulation attacks.

We set Nodes 4, 11, and 23 as the message manipulation attackers. Assume that they broadcast the invalid time message $\tau_{i}\left(t\right)+\omega$ every five cycles, where ω denotes the attack power and is randomly selected within 0–0.01 s. With the existence of malicious nodes, the safe nodes in MTS estimate $\alpha_{ij}$ according to invalid time messages, and then the updating rules will be destroyed. For SMTS and NiSTS, they update the clock based on the uniqueness of relative clock skew. Invalid time messages could not pass their detection process. Hence, the logical clock skew in MTS could not converge under messages manipulation attacks. SMTS and NiSTS are robust against the message manipulation attacks, as shown in Figure 8. Similar to the above analysis, NiSTS updates its logical clock after computing the relative clock skew with two neighbors at least three times, whereas SMTS only needs to compute the relative clock skews with one neighbor at least two times, so the convergence time of NiSTS is longer than that of SMTS. Meanwhile, SMTS can isolate the malicious nodes to synchronize fewer nodes in IWSNs, which would accelerate its convergence.

**Figure 9:**
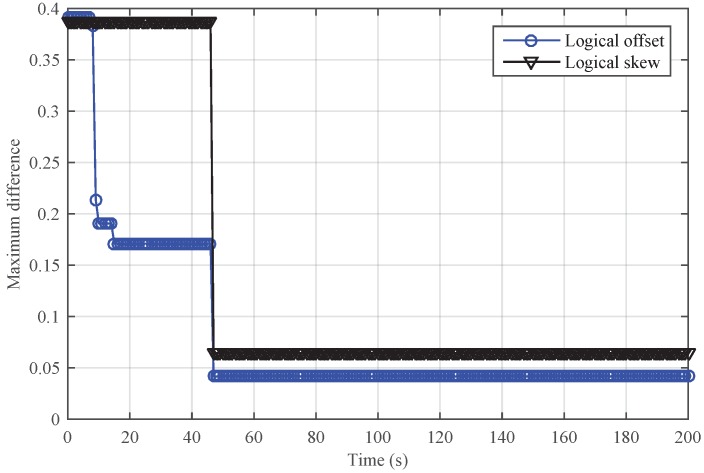
Performance of SMTS under Sybil attacks.

**Figure 10:**
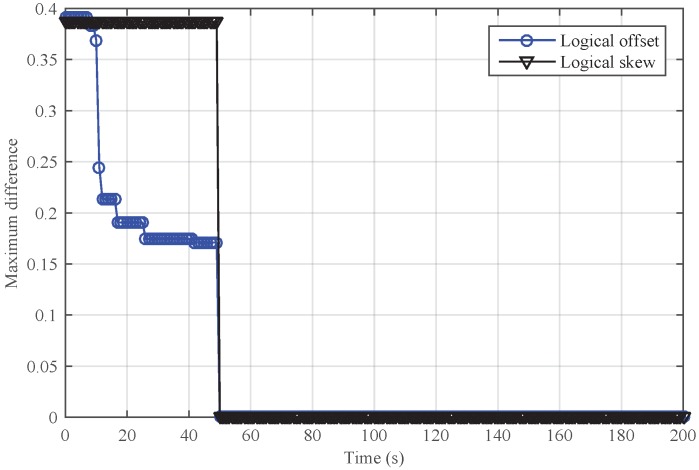
Performance of NiSTS under Sybil attacks.

Figure 9 and Figure 10 show the performance of SMTS and NiSTS under Sybil attacks, respectively. Here, we also set Nodes 4, 11 and 23 as the Sybil attackers who broadcast an invalid time message every five cycles with a false identity. The identity of the attackers is randomly selected from their neighbors. Under Sybil attacks, the safe nodes disguised by the attacker (namely, the suspicious nodes) would be regarded as malicious nodes in SMTS, and then SMTS would breaks up with the suspicious nodes. For NiSTS, it will not disrupt the communication with the suspicious nodes. The synchronization process continues when the messages of suspicious nodes pass the detection mechanism. Therefore, NiSTS is effective under Sybil attacks but the maximum errors of the logical skew and the logical offset of SMTS converge to two constant values, respectively. From the results in Figure 9, we conclude that the logical clock error of SMTS initially decreases but then linearly increases, i.e., Theorem 1 is indirectly approved. Meanwhile, the convergence time of NiSTS is slightly longer than that of SMTS, as the updating start time of NiSTS is later that of SMTS.

**Figure 11:**
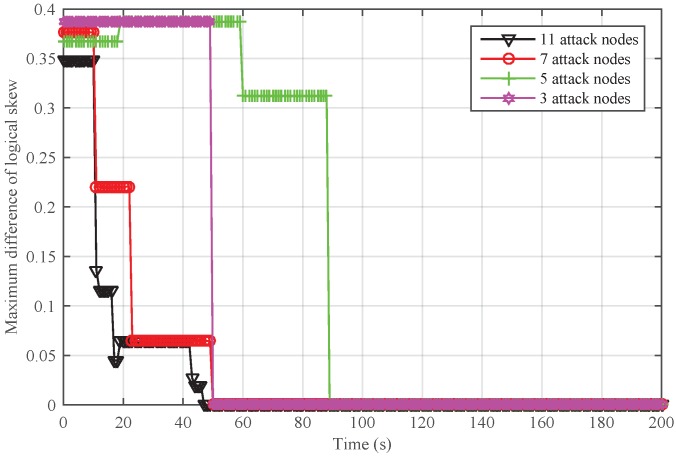
The performance of NiSTS against different numbers of Sybil attackers that are generated from the inside network.

To further analyze the scenario that Sybil attackers are generated from the inside network, we adjusted the number of attack nodes and safe nodes for a given network. The total number of nodes was set to 30. We randomly chose 3, 5, 7, and 11 attackers from the network. In Figure 11, it can be seen that NiSTS could also realize time synchronization and may defend against more attackers.

**Figure 12:**
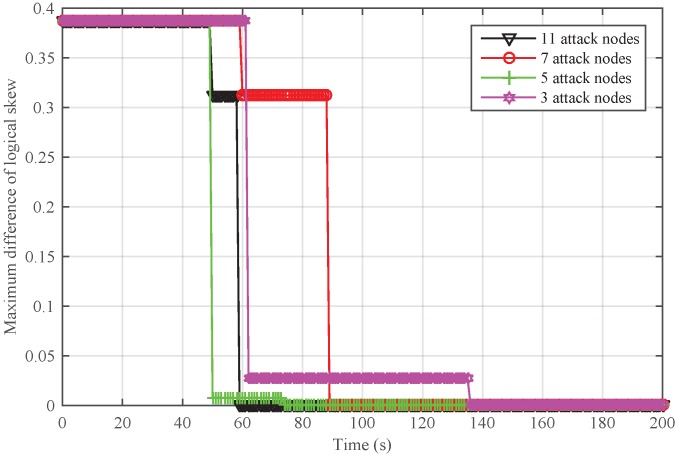
Maximum difference of logical skew for NiSTS against different numbers of Sybil attackers for a fixed network.

**Figure 13:**
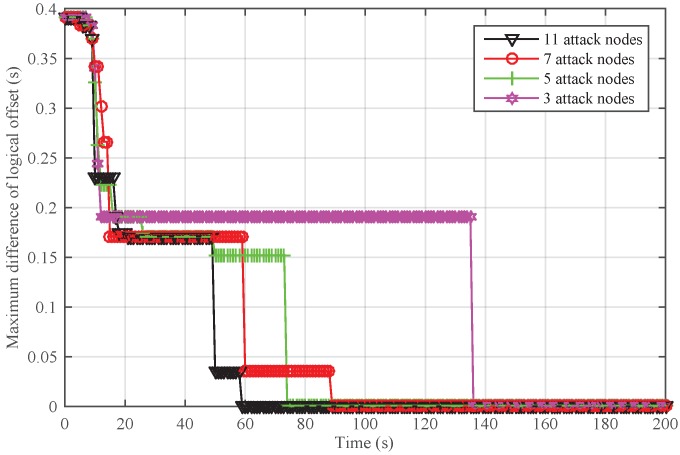
Maximum difference of logical offset for NiSTS against different numbers of Sybil attackers for a fixed network.

Next, we conducted simulations to reveal the impact of the number of Sybil attacker on NiSTS. The probability factor ε is 0.2. Thus, the maximum number of Sybil attackers is 11. We considered four scenarios with 3, 5, 7, and 11 attackers, respectively. The total number of safe nodes in each scenario was set to 30. The initial clock parameters and location information of the safe nodes in these four network scenarios are same. To better illustrate the effectiveness of NiSTS, the attack period was reduced to two cycles. The attacker power was also randomly selected within 0–0.01 s. Figure 12 and Figure 13 demonstrate that adding attack nodes has no influence on the convergence of NiSTS.

As the attackers randomly select identities from their neighbors, the synchronization path is interfered. Thus, the convergence time of NiSTS for a given network under Sybil attacks is not a constant value. Of course, fewer Sybil attackers may bring longer convergence time. In Figure 12 and Figure 13, it can be seen that there is no relationship between the convergence time and the number of Sybil attackers. Therefore, the simulation results reveal that the convergence time of NiSTS is not sensitive to the number of attackers with a given ε.

Figure 6, Figure 8 and Figure 10 indicate that NiSTS is not sensitive to the attack types.

## 6. Conclusions

Secure time synchronization is crucial for many applications in IWSNs. This paper studies time synchronization under Sybil attacks in IWSNs. The simulation results and theoretical analysis are given to show that the logical clock error of SMTS linearly increases at last with probability under Sybil attacks. We have presented a new time synchronization scheme that defends against Sybil attacks: the node-identification-based secure time synchronization (NiSTS) protocol is constructed with two phases. The first phase consists of message filtering rather than node isolation. The second phase involves updating the logical clock parameters based on an existing time synchronization algorithm. In this paper, we adopt the MTS protocol due to its fast convergence and simultaneous compensation of the clock skew and offset. The theoretical analysis and simulation results reveal that the convergence time of NiSTS is not sensitive to the number of Sybil attackers with a given probability factor and could defend against both the message manipulation attacks and Sybil attacks. Future directions include extending the NiSTS to complicated link models and other time synchronization algorithms. As the node density is relative to the effectiveness of detection process, we would investigate and optimize the network deployment to guarantee the effectiveness of NiSTS in future works.

## Figures and Tables

**Table 1:** Notation definitions.

Symbol	Definition
$\tau_{i}\left(t\right)$	the hardware clock reading of node *i* at time *t*;
$\alpha_{i}$	the hardware clock skew of node *i*;
$\beta_{i}$	the hardware clock offset of node *i*;
${\hat{\tau }}_{i}\left(t\right)$	the logical clock reading of node *i* at time *t*;
${\hat{\alpha }}_{i}\left(t\right)$	the skew compensation parameter of node *i* at time *t*;
${\hat{\beta }}_{i}\left(t\right)$	the offset compensation parameter of node *i* at time *t*;
*n*	the number of safe nodes;
*m*	the number of attackers;
$\alpha_{ij}\left(k\right)$	the relative clock skew of node *i*, *j* at the *k*th iteration;
$t_{k}^{+}$	the time after updating at time $t_{k}$;
$V_{s}$	the set of safe nodes;
$V_{sus}$	the set of suspicious nodes;
$V_{valid}$	the set of valid nodes;
$V_{iso}$	the set of isolated nodes.
